# Repeated administration of the NSAID meloxicam alters the plasma and urine lipidome

**DOI:** 10.1038/s41598-019-40686-4

**Published:** 2019-03-13

**Authors:** Sol M. Rivera-Velez, Liam E. Broughton-Neiswanger, Martin Suarez, Pablo Piñeyro, Jinna Navas, Sandy Chen, Julianne Hwang, Nicolas F. Villarino

**Affiliations:** 1 Program in Individualized Medicine, Department of Veterinary Clinical Sciences, College of Veterinary Medicine, Washington State University, Pullman, 99164 WA United States; 2 Department of Veterinary Clinical Sciences, College of Veterinary Medicine, Washington State University, Pullman, 99164 WA United States; 3 Veterinary Diagnostic Laboratory, College of Veterinary Medicine, Iowa State University, Ames, 1134 IA United States

## Abstract

Non-steroidal anti-inflammatories (NSAIDs), such as meloxicam, are the mainstay for treating painful and inflammatory conditions in animals and humans; however, the repeated administration of **NSAIDs** can cause adverse effects, limiting the long-term administration of these drugs to some patients. The primary aim of this study was to determine the effects of repeated meloxicam administration on the feline plasma and urine lipidome. Cats (n = 12) were treated subcutaneously with either saline solution or 0.3 mg/kg body weight of meloxicam daily for up to 31 days. Plasma and urine lipidome were determined by LC-MS before the first treatment and at 4, 9 and 13 and 17 days after the first administration of meloxicam. The repeated administration of meloxicam altered the feline plasma and urine lipidome as demonstrated by multivariate statistical analysis. The intensities of 94 out of 195 plasma lipids were altered by the repeated administration of meloxicam to cats (*p* < 0.05). Furthermore, we identified 12 lipids in plasma and 10 lipids in urine that could serve as biomarker candidates for discriminating animals receiving NSAIDs from healthy controls. Expanding our understanding about the effects of NSAIDs in the body could lead to the discovery of mechanism(s) associated with intolerance to NSAIDs.

## Introduction

Better pain management is one of the five federal responses of the Department of Health and Human Services to the devastating opioid epidemic occurring in the United States^1^. Indeed, new strategies for controlling chronic pain are urgently needed to tackle this epidemic. Non-steroidal anti-inflammatory drugs (NSAIDs), such as meloxicam, could be used to ameliorate chronic pain while helping to reduce the use of addictive opioids. However, chronic administration of these drugs can cause gastrointestinal and renal damage in some patients, limiting the therapeutic options of such patients in need of pain and inflammation control. Expanding our understanding about the effects of NSAIDs in the body could lead us to reveal the mechanism(s) associated with intolerance to NSAIDs; in turn, this will enable to discover new drug targets and/or therapeutic strategies for the optimal control of pain and inflammation. Furthermore, identification of drug-induced changes in lipids may result in the discovery of novel substances that could become candidate markers for monitoring the use of NSAIDs in patients. Metabolic markers could assist, after proper validation, the categorization of patients as having a high or low risk of suffering adverse effects^2^, which would help establish new targeted treatment strategies, thereby enhancing patient care.

NSAIDs have a pleiotropic effect in the body, influencing cell function and metabolism in most tissues^3–6^. NSAIDs can alter lipid metabolism, in fact, one of their primary mechanisms of action is related to intracellular lipid pathways^4^. *In vitro* studies have reported that NSAIDs induce cellular fatty acid desaturation, alter cellular phospholipid components, and elevate the level of free fatty acids^4^. Considering all this evidence, we hypothesize that the repeated administration of the NSAID meloxicam alters the plasma and urine lipid content in cats.

Because lipids are diverse and abundant molecules^7,8^, we tackled this hypothesis by profiling the total repertoire of lipids in plasma and urine using an untargeted lipidomic approach in cats treated repeatedly with meloxicam. The study of lipids in plasma and urine provides a powerful non-invasive means for assessing the status of cellular metabolic processes occurring in the body’s tissues. The application of lipidomics in a number of diseases, including diabetes^9,10^, cardiovascular diseases^11^, and other inflammatory processes^12^ has already provided characteristic lipid signatures and mechanistic insights into disease processes^13,14^. The resulting lipidome profile is analogous to a chemical fingerprint left behind by NSAID-induced alterations to cellular processes. Therefore, as a second objective, we sought to identify lipids in plasma and urine that could be assessed prospectively as putative biomarkers for monitoring the effect of NSAIDs.

## Results

All study animals were clinically healthy according to physical examinations, cellular blood counts and blood chemistry panels prior to administration of treatments. Once treatments were initiated, cats in the control group remained healthy for the duration of the study. One cat in the control group vomited once on day 4 after the first drug administration. In the meloxicam treated group, the cats’ body-weights and condition scores were relatively stable, except for one cat (M_5) whose body weight was reduced by ~7%, likely due to a decrease in the food intake. During the period of sample collection (17 days), 5 out of 6 cats in the meloxicam group vomited 2 to 11 times. One cat vomited a total of 15 times but no more than once a day; however, her food intake, body weight, and condition scores were consistent with pre-treatment values.

Prior to administration of the treatments, all cats had comparable serum concentrations of BUN and creatinine, all within the normal range (Supplementary Figs S1 and S2). In the control group, the serum concentration of creatinine and BUN remained relatively steady. In the meloxicam group, the mean serum creatinine concentration was comparable to that observed in the control group up to 9 days following the first administration of meloxicam. After day 9, a noticeable abnormal increment of the mean serum concentration of creatinine and BUN occurred (Supplementary Figs S1 and S2).

### Feline lipidome

One hundred ninety-five known lipids were detected in positive ion mode from the extracted plasma and urine samples. We limited the statistical analyses to the dataset obtained with the positive mode electrospray (ESI) because this mode provided more annotated lipids than ESI (−). These lipids included fatty acyls, glycerolipids, glycerophospholipids, sphingolipids and sterol lipids (Supplementary Table S1).

### Changes in the plasma lipidome induced by the repeated administration of meloxicam to cats

**Figure 1:**
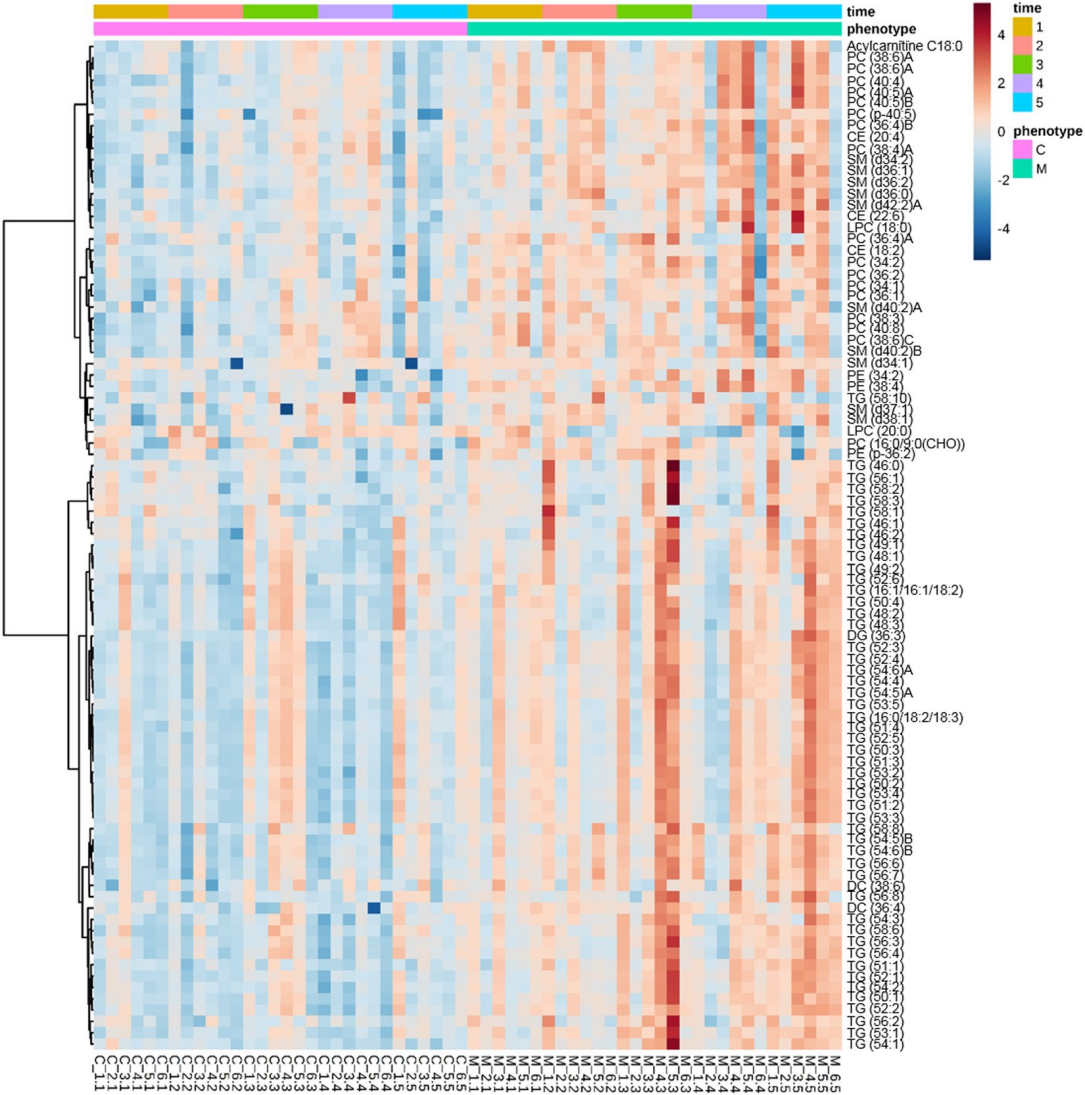
Heatmap clustering lipids in plasma from meloxicam-treated (n = 6) and saline-treated (n = 6) cats before (time 1) and after the administration of saline or meloxicam at 0.3 mg/kg every 24 h for up to 17 days (times 2 to 5). For simplicity, the figure only displays lipids with intensities that were different (*p* < 0.05) based on ANOVA. The higher the signal intensity of a lipid, the more red in the heatmap gradient colors. The lower the signal intensity of a lipid, the bluer in the heatmap gradient colors. Each row corresponds to a lipid. Each column represents each cat at a given sampling time. Data is clustered based on phenotype.

Two-way ANOVA was conducted to identify the lipids in plasma whose signal intensity was different between meloxicam- and saline-treated cats. The intensities of 94 out of 195 lipids were altered by the repeated administration of meloxicam to cats (*p < *0.05) (Supplementary Table S2). A heatmap including lipids that were different as per ANOVA, revealed noticeable differences in lipid intensities between meloxicam- and saline-treated cats and these differences were more evident over time (Fig. 1). The heatmap color gradients for the saline-treated cats tended to be relatively constant over time. In contrast, the color gradients in plasma from meloxicam-treated cats were darker over time, reflecting higher lipid intensities.

### Comparison between groups: control cats and cats treated with meloxicam, using PLS-DA score plots

To visualize the clustering of tested samples, a PLS-DA model was constructed. The PLS-DA model showed a good prediction (Q^2^) and fitting performance (R^2^) at sampling times 2, 4 and 5 (Supplemental Fig. S3). Q^2^ values obtained for time 1 (before administration of meloxicam) and time 3 indicated that PLS-DA was not a good classification model. Therefore, this model was not applied to the data set obtained for sampling time 3. Regarding the classification of the groups at time 1, a low Q^2^ value was expected for the model since this time point is prior to the first dose of meloxicam. Overall, PLS-DA score plots for time points 2, 4 and 5 (after the administration of meloxicam at 4, 13 and 17 days, respectively) showed a distinct separation between meloxicam- and saline-treated cats. The principal component 1 and 2 explained together 56.0%, 34.5% and 52.9% of the variability for time points 2, 4 and 5, respectively (Supplementary Fig. S3).

### Identification of plasma lipids that could serve as biomarker candidates to distinguish meloxicam- and saline-treated cats

**Figure 2:**
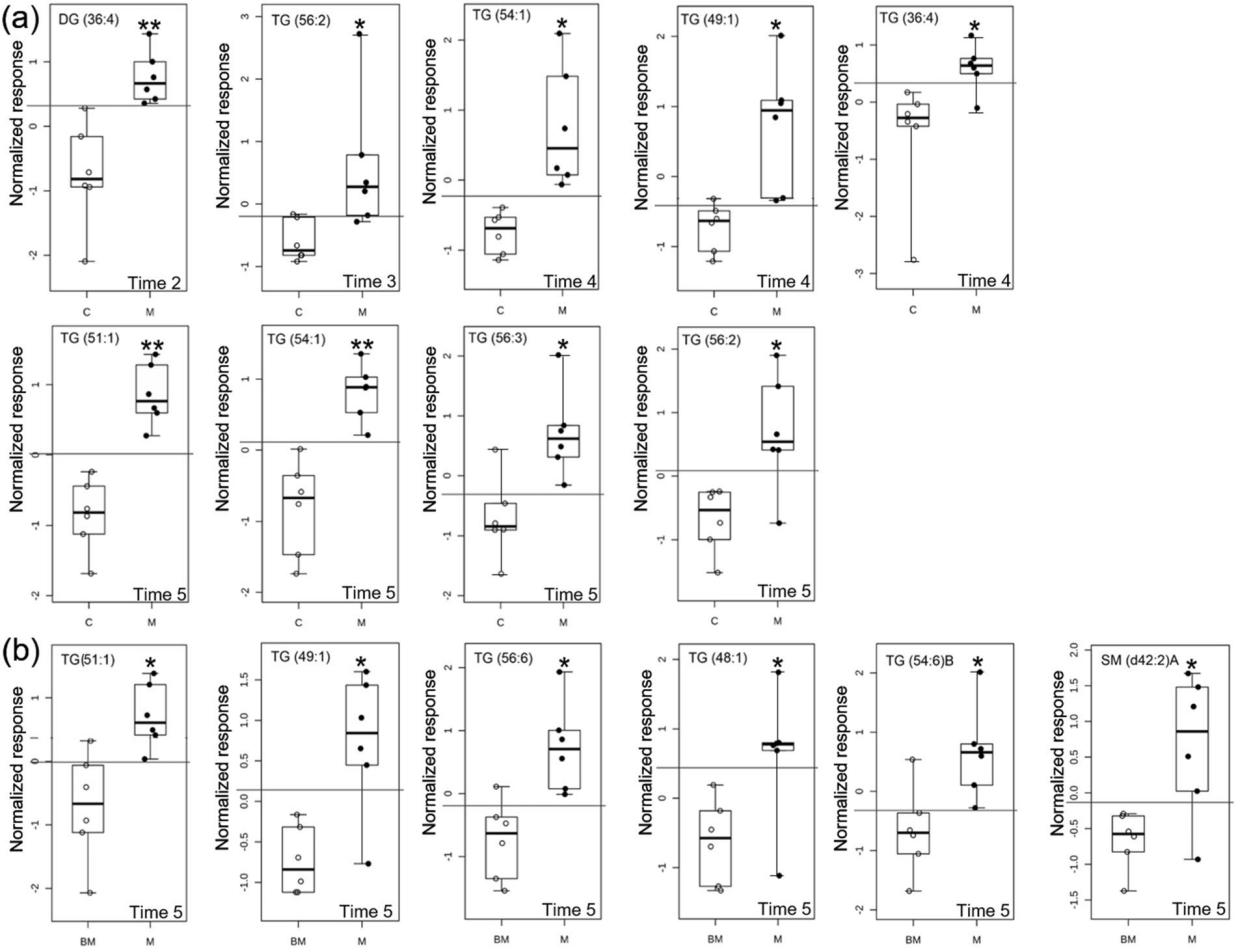
Comparison of the normalized signal intensity of lipids in plasma that were identified as biomarker candidates for discriminating **(a)** meloxicam-treated (M) (n = 6) and saline-treated (C) cats (n = 6); and **(b)** baseline (BM) and meloxicam group (M) at different times after the administration of saline or meloxicam at 0.3 mg/kg every 24 h for up to 17 days (times 2 to 5). The lines in the ROC boxes represent the median intensity value for each lipid; the upper and lower boundaries of the box indicate the 75th and 25th percentiles, respectively; the upper and lower whiskers represent the maximum and minimum values. The horizontal lines in the ROC boxes indicate the highest sensitivity and specificity in separating control-  from meloxicam-treated animals. *p < 0.05 and **p < 0.001.

In order to identify plasma lipids that could help to discriminate meloxicam-from saline-treated cats, we combined three different approaches: (1) variable importance in projection, obtained from the PLS-DA model, (2) receiver operating characteristic curves and, (3) random forest analysis. The criteria of acceptance to consider a lipid as biomarker candidate for each approach were (i) a ROC AUC > 0.85, (ii) a mean decrease accuracy ≥0.004 and (iii) a VIP ≥ 1 value for the receiver operating characteristic, random forest and PLS-DA analyses, respectively. Only those lipids that meet the selection criteria for the three approaches at each sample time were selected as biomarker candidates, minimizing the likelihood of capturing irrelevant lipids. Supplementary Table S3 and Fig. S4 show the list of lipids which fulfilled the criteria of acceptance for the variable importance in projection from the PLS-DA model and the random forest, respectively. Six lipids, DG (36:4), TG (56:2), TG (54:1), TG (49:1), TG (51:1) and TG (56:3), were identified as potential biomarker candidates to discriminate saline and meloxicam-treated cats. In the meloxicam-treated cats the intensity of these lipids increased from 1.6 to 4.1-fold (Supplementary Table S4). Figure 2 shows the time point at which each of these lipids was identified.

### Identification of biomarker candidates in plasma within the meloxicam-treated cats

In order to identify lipids that could be used to monitor patients under meloxicam treatment, we evaluated the lipidome changes from baseline in the meloxicam-treated cats. Six lipids, TG (51:1), TG (49:1), TG (56:6), TG (48:1), TG (54:6)B and SM (d42:2)A were identified as potential biomarker candidates for monitoring meloxicam-treated cats. These lipids changed their signal intensity from baseline as compared to time point 5 (Fig. 2). The intensity of the lipids increased between 1.8- and 2.5-fold from baseline. The AUC ROC, fold change, and mean decrease accuracy values obtained to select these lipids are reported in the Supplementary Table S4 and Fig. S5.

### Changes in the urine lipidome induced by the repeated administration of meloxicam to cats

**Figure 3:**
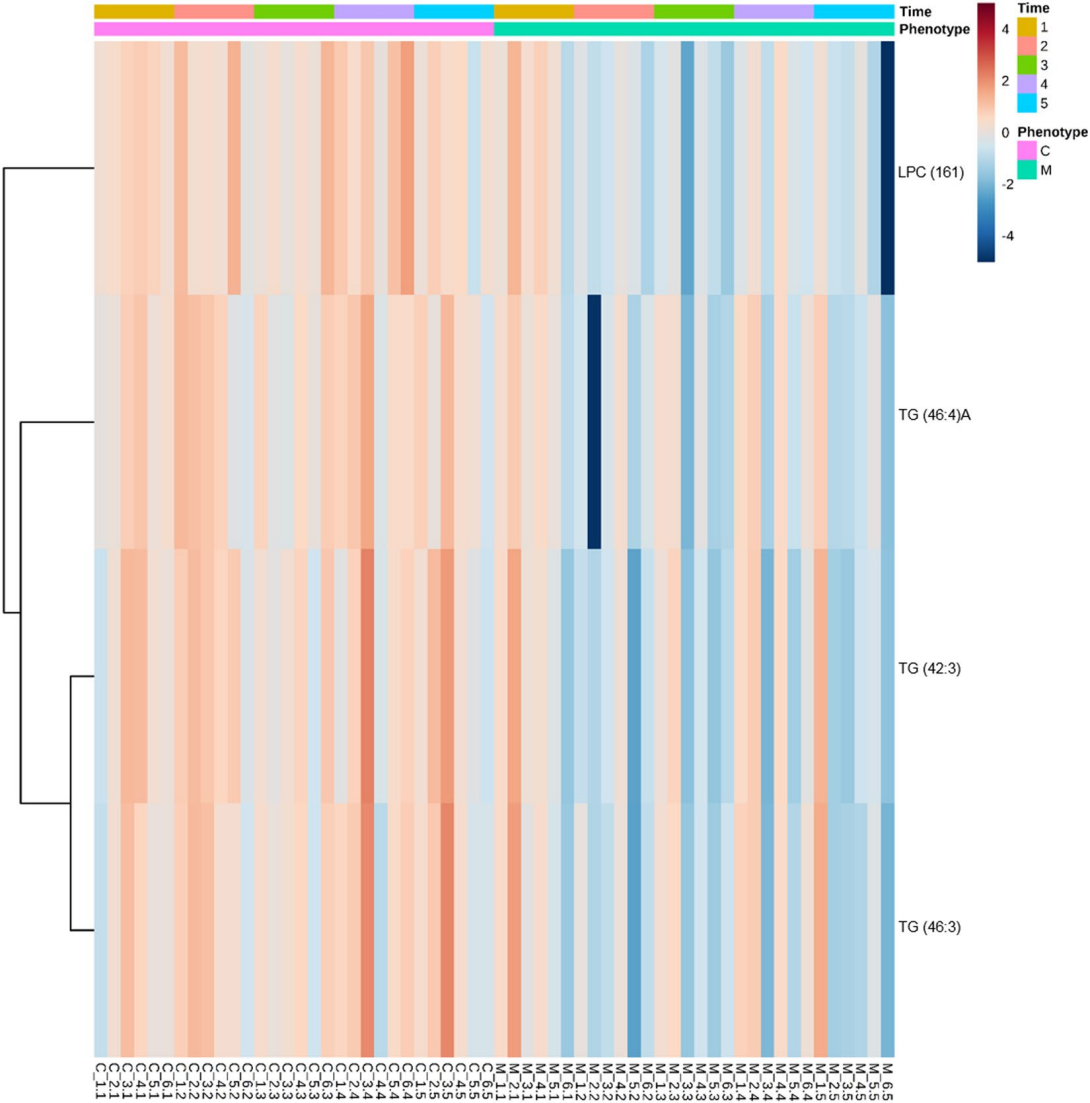
Heatmap clustering lipids in urine from meloxicam-treated (n = 6) and saline-treated (n = 6) cats before (time 1) and after the administration of saline or meloxicam at 0.3 mg/kg every 24 h for up to 17 days (times 2 to 5). For simplicity, the figure only displays lipids with intensities that were different (*p* < 0.05) based on ANOVA. The higher the signal intensity of a lipid, the more red in the heatmap gradient colors. The lower the signal intensity of a lipid, the bluer in the heatmap gradient colors. Each row corresponds to a lipid. Each column represents each cat at a given sampling time. Data is clustered based on phenotype.

Two-way ANOVA was conducted to identify the urine lipids whose signal intensity was different between meloxicam- and saline-treated cats. LPC (16:1), TG (46:4)A, TG (42:3) and TG (46:3) were statistically different in the meloxicam and saline-treated cats (*p* = 0.002, 0.017, 0.012 and 0.028, respectively). In addition, the heatmap revealed that the lipid intensities in the meloxicam-treated cats tended to decrease over time compared to the control group (Fig. 3).

### Identification of lipids in urine that could serve as biomarker candidates to distinguish meloxicam- and saline-treated cats

**Figure 4:**
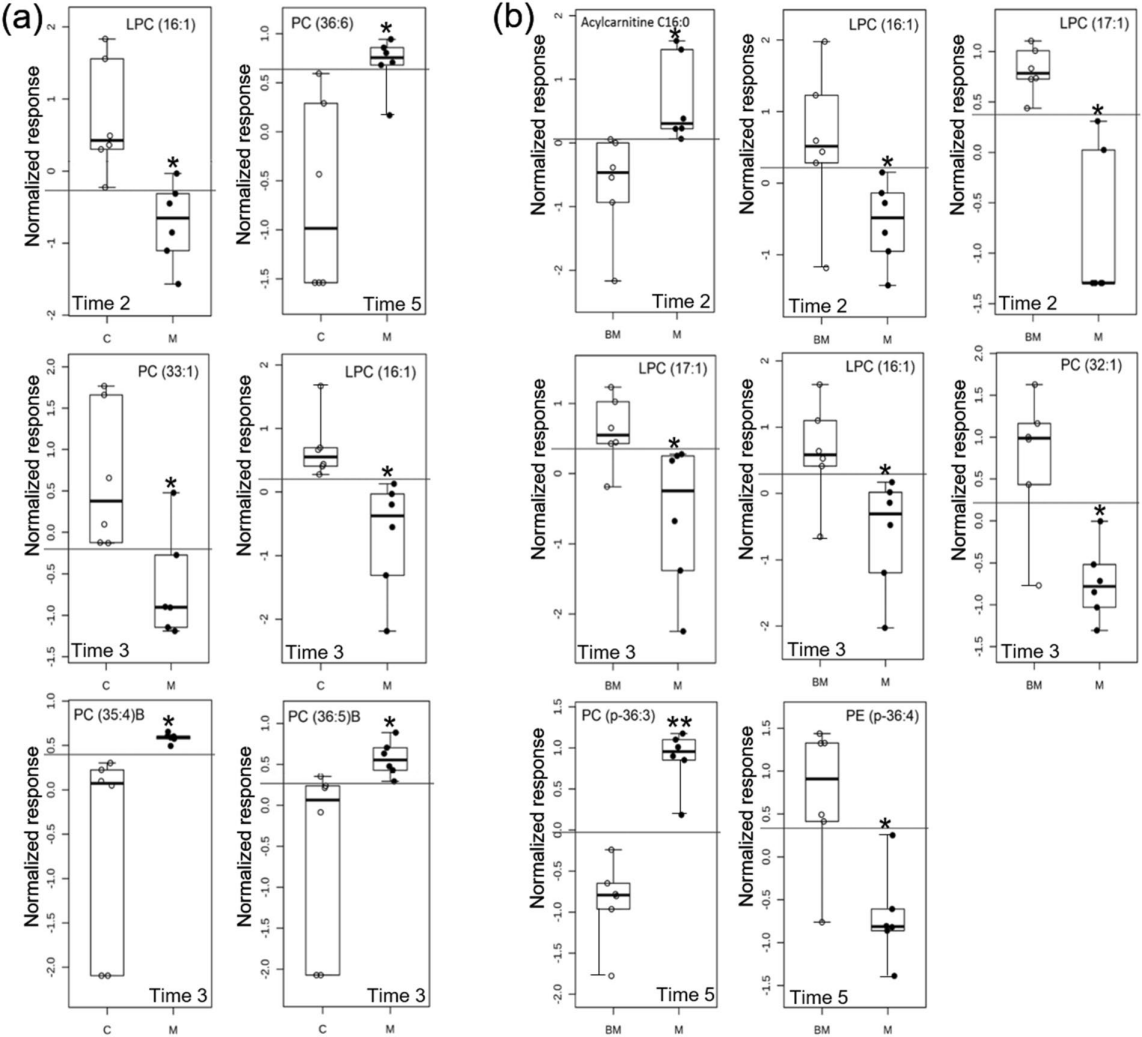
Comparison of the normalized signal intensity of lipids in urine that were identified as biomarker candidates for discriminating (**a**) meloxicam-treated (M) (n = 6) and saline-treated (C) cats (n = 6); and (**b**) baseline (BM) and meloxicam group (M) at different times after the administration of saline or meloxicam at 0.3 mg/kg every 24 h for up to 17 days (times 2 to 5). The lines in the boxes represent the median intensity value for each lipid; the upper and lower boundaries of the box indicate the 75th and 25th percentiles, respectively; the upper and lower whiskers represent the maximum and minimum values. The horizontal lines in the ROC boxes indicate the highest sensitivity and specificity in separating between control and meloxicam animals. *p < 0.05 and **p < 0.001.

The results of the PLS-DA for the urine lipidome are not described in this section because this model did not fit the data (data not shown). Consequently, the selection of the biomarker candidates was based only on the results obtained with the receiver operating characteristic and the random forest analyses (Supplementary Table S5 and Fig. S6). Applying the same criteria used for the plasma lipidome to select the biomarker candidates, five lipids, LPC (16:1), PC (33:1), PC (35:4)B, PC (36:5)B and PC (36:6), discriminated meloxicam-treated from saline-treated cats. Putative biomarkers for distinguishing meloxicam- and saline-treated cats at different time points are shown in Fig. 4. In general, the abundances of LPC (16:1) and PC (33:1) were higher in the meloxicam group than those in the control group. However, the abundances of PC (35:4)B, PC (36:5)B and PC (36:6) were lower in the meloxicam group than those in the control group. Notably, the LPC (16:1) was selected for time points 2 and 3 (Fig. 4).

### Identification of biomarker candidates in urine within the meloxicam-treated cats

Six lipids were identified as potential biomarker candidates in urine samples from meloxicam-treated cats. Comparison of the signal intensity of the biomarker candidates in the meloxicam group before and after the administration of meloxicam are shown in Fig. 4. The abundances of acylcarnitine (16:0) and PC (p-36:3) were higher after the administration of meloxicam. On the contrary, the abundances of LPC (17:1), LPC (16:1), PC (32:1) and PE (p-36:4) were lower in the meloxicam group after the administration of the drug  relative to baseline. Time points at which each biomarker candidate was selected are displayed in Fig. 4. The AUC ROC and mean decrease accuracy values obtained for each biomarker candidate are reported in Supplementary Table S5 and Fig. S7.

Concentrations for all the potential biomarker candidates at each time point and for each matrix can be seen in Supplementary Table S6.

## Discussion

In this study, we evaluated the effect of repeated meloxicam administration on the plasma and urine lipidome in domestic cats. The results of this study reveal that repeated administration of the NSAID meloxicam altered the feline plasma and urine lipidome in young-adult cats (Figs 1 and 3). In plasma, the lipidome changed following the start of meloxicam administration. The heatmap suggests a time-dependent effect of meloxicam on the plasma lipidome in cats (Fig. 1). The mechanism of lipid changes observed in the study is unknown. The endoplasmic reticulum is the main site of lipid synthesis, degradation and remodeling^15–18^. Perturbation of endoplasmic reticulum homeostasis can influence the lipid levels and profiles in the lipidome^19^. Recent investigations reveal that the NSAID indomethacin^20,21^ and diclofenac^22^ can cause endoplasmic reticulum stress, which could alter the lipid machinery. Oxicam-NSAIDs, such as meloxicam, are also active in the cellular endoplasmic reticulum^23^. Changes in the endoplasmic reticulum lipid metabolism caused by meloxicam could explain, at least in part, our findings. However, the effect of meloxicam and other NSAIDs on lipid synthesis, degradation, remodeling and the impact on the lipidome remains unknown and deserves to be studied.

The specific source of the lipidome changes in plasma could reflect the meloxicam-induced effects on various organs. The repeated administration of 0.3 mg/kg of meloxicam to cats is known to damage renal tubules and decrease renal filtration^24^. As expected, cats in the meloxicam group had increased serum BUN and creatinine concentrations (Supplementary Figs S1 and S2). The increment in serum creatinine and BUN concentrations are consistent with acute kidney injury in cats, according to the International Renal Interest Society^25^, suggesting that the repeated administration of meloxicam altered kidneys’ filtration. Damaged kidneys may have contributed to the lipidome changes detected in the plasma of the meloxicam-treated cats. Of note, several studies have demonstrated remarkable elevation of serum triacylglycerides in patients with different kidney diseases compared to the healthy controls^26–28^. In this study, we found significant increases in the plasma levels of triacylglycerides in meloxicam-treated cats (Fig. 2). However, it is unlikely that these changes are the sole result of the effect of meloxicam on the kidneys. It is important to remark that most of the cats (5/6) in the meloxicam-treated group vomited several times during the sample collection period. As far as the authors’ knowledge no studies have reported lipidomic changes caused by vomits in any species yet. However, it is possible that vomits could have had an impact on the lipidome changes, which also warrants further investigation.

As observed in plasma, the repeated administration of meloxicam also disturbed the urine lipidome (Fig. 3). Urine samples can be considered a distal liquid biopsy of the kidneys^29^, thus it is reasonable to speculate that some of the changes in the urine lipidome reflect effects of meloxicam on this organ. Support to this assumption is also provided by the fact that the lipid changes identified in urine were not the same as those identified in plasma; indeed a future study specifically designed to investigate these differences will be required. Notably, in this study, 9 out of 10 biomarker candidates identified in the urine were glycerophosphocholines and glycerophosphoethanolamines, suggesting a profound disturbance of glycerophospholipid metabolism caused by the repeated administration of meloxicam to cats. Glycerophospholipids are the main components of the cell membranes and play a major role in cell signaling, membrane anchoring, and substrate transport^30^. Previous studies have provided evidence that glycerophospholipid metabolism is altered in patients with kidney disease^31,32^. For instance, urinary phosphatidylcholines concentrations were significantly higher both in glomerulonephritis and tubulointerstitial injury than in healthy controls^32^. Similarly, several renal lysophosphatidylcholines and ether-linked phospholipids have been reported as potential biomarkers for acute kidney injury^31^.

With lipidomic changes in plasma and urine following the repeated administration of meloxicam confirmed, the next step was to identify the lipids in plasma and urine that discriminate meloxicam-treated cats from saline-treated cats. The identification of feline-specific pre-clinical biomarkers of NSAID-induced toxicity would be extremely valuable for optimizing individualized drug therapies. Non-invasive biomarkers would assist clinicians make therapeutic decisions according to individual needs; for example, the selection of optimal dose intervals and the extent of washout periods between drug administrations, and/or the selection of intermittent therapy versus daily administration; thus, maximizing the benefits of NSAIDs and minimizing the risk of adverse effects. In order to minimize the likelihood of capturing irrelevant lipids, the selection of potential biomarkers was rigorously scrutinized by applying three approaches: the receiver operating characteristic curve, random forest, and PLS-DA analyses^33–35^. In this study, we identified 6 lipids in plasma and 5 in urine as putative biomarkers to discriminate meloxicam-treated from saline treated-cats (Figs 2 and 4). All these lipids could be further studied using targeted lipidomics to confirm their potential value as biomarker candidates for monitoring the administration of NSAIDs in patients over time.

From the clinical standpoint, it would be valuable to monitor the response of patients before and after treatment over time, particularly when patients receive repeated treatments over long periods of time. Therefore, we evaluated the lipidomic changes in plasma and urine in meloxicam-treated cats by comparing post-treatment vs. pre-treatment (baseline) lipidome. In plasma from meloxicam-treated cats, we identified several lipids able to discriminate cats pre- and post-meloxicam (Figs 2 and 4). However, only TG (49:1) and TG (51:1) were identified as putative biomarkers when the lipidome was compared between meloxicam- and saline-treated cats and within the meloxicam group (post-treatment vs baseline lipidome) (Fig. 2). In urine, LPC (16:1) was the only lipid that was identified as biomarker candidate to discriminate meloxicam- vs saline-treated cats and post- and pre-meloxicam administration (Fig. 4). The physiological roles of the above-mentioned lipids in the body have yet to be elucidated.

In this study, the NSAID selected for investigation was meloxicam but it would be interesting to assess whether the observed lipidome changes are also caused by other NSAIDs. In order to expand our understanding about the effects of NSAIDs on the plasma and urine lipidome, it is necessary to evaluate the potential impact of genders, age other environmental conditions and species (e.g. other animal models). It is noteworthy that this study was not specifically designed to discover biomarkers because it was unknown if NSAIDs had any impact on the lipidome. However, the information generated here is key for expanding this new research line. The design of multiple-dose-multiple-NSAID studies could assist to discover biomarker candidates for monitoring the administration of NSAIDs. Most of the lipids that were able to distinguish saline-treated from meloxicam-treated cats belong to the lipid classes of PC, TAG, FA, and SM. Importantly, all these lipids can be affected by dietary intake. Even though there are lipid-based biomarkers used clinically that are affected by dietary intake (e.g., cholesterol and TG for cardiovascular disease), the effect of dietary intake on the plasma and urine lipidome need to be considered as a critical factor for discovering novel lipid-based biomarkers. Dietary intake can influence the inter- and intra-individual variability in the substance concentrations. A relative large within- and between-subject variation in lipid concentrations can reduce the robustness and clinical utility of substances as potential biomarkers. Before establishing the clinical utility of novel lipids, studies should be specifically designed to understand if lifestyle factors (e.g., diet intake, habit exercise, obesity, etc.), ancillary pathological conditions or certain drugs affect the potential value of lipids as biomarkers for diagnosing, or monitoring the used of NSAIDs in cats and any other species. This study provides novel information, nevertheless, the small sample size used in this study highlights its preliminary nature. For both, multidimensional statistical power analyses of urine and plasma lipidome suggest that our study obtained statistical significance for a subset of lipids. Future studies should include a more heterogeneous population with a larger sample size because other lipids could become statistically significant (~200 cats per group for plasma and >1000 cats for urine (Supplementary Fig. S8)). Future assessment of the lipids identified as potential biomarkers would also require larger sample sizes (from 4 to 75 depending on the lipid and matrix; Supplementary Figs S9–S12). It is important to highlight that there is currently no single analytical method available to capture the full lipidome in a single analysis. In our study, there were only 195 known lipids while ~1100 compounds were unknown in plasma and urine. Thus, the lipids identified in this study represent a small fraction of the lipidome. Identification of each uncharacterized metabolite will require to combine different analytical platforms such as LC-MS^36^, GC-MS^36^, shutgun^37^, preparative scale isolation for NMR studies or extensive chemical synthesis to enable structural comparisons using tandem mass spectrometry^38^. Expanding the knowledge about the lipidome would reveal effects of NSAIDs that were not captured in this study.

## Conclusion

This is the first report to identify lipidomic changes in urine and plasma caused by the repeated administration of meloxicam to healthy young-adult cats. The repeated administration of meloxicam left behind a feline lipidic fingerprint, which provides valuable and novel information, as well as laying the foundation for continued research into NSAID therapy, lipidomics, and biomarker discovery. Nonetheless, it is important to stress that the lipids identified in this study are putative biomarkers and should not be used for monitoring NSAID-based therapies until the clinical value of these lipids has been verified and validated.

## Materials and Methods

### Study population and inclusion criteria

The Washington State University (WSU) Institutional Animal Care and Use Committee approved all study procedures before their initiation (ASAF#04915). All methods were performed in accordance with the relevant guidelines and regulations. Twelve female clinically healthy intact adult (1-1.5 years old) purpose bred cats (2.5–3.8 Kg) were obtained from a USDA-licensed commercial breeder (Nutrition and pet Care Center UC DAVIS, Davis, USA). The primary enrollment criteria were no signs of illness or infection and no history of drug treatment within 30 days before initiation of the study. Every cat was given a baseline complete history, physical examination, routine blood chemistry panel and urinalysis. All cats were vaccinated for rabies before being transferred to WSU.

### Animal management and monitoring

Cats were acclimated to the new housing environment at least 10 days before starting the study. Each cat was housed separately in cages. The room was temperature (70–73 °F), humidity (25–35%) and 12 h light/dark cycle controlled. The cats had free access to drinking water and food (Purina Cat Chow Indoor Formula) throughout the study. Fresh water and food were changed every day. Cages were cleaned, and litter boxes were changed every day. Cats were examined physically at least twice daily during the entire study duration to rule out possible health problems.

Following the acclimation period, vascular access ports (VAPs, petite size; Le Port Companion Port, Norfolk Vet, Skokie, IL, USA) were implanted subcutaneously seven days before starting the administration of the treatments following standard procedures as recommended by the manufacturer. The VAPs were implanted aseptically in the jugular vein. The VAPs were maintained with a heparinized saline solution (100 I.U./mL, 0.7 mL each time).

### Controlled randomized experimental design

Following the VAPs implantation, cats were allocated randomly to 2 experimental groups; control (n = 6) and meloxicam (n = 6) groups. The sample size of this study was based on the minimum number of experimental units used in exploratory metabolomics studies^39^. Randomization of the treatments was done using R studio (V 3.1.1). All cats were treated subcutaneously with a volume of saline at 0.1 mL/kg body weight every 24 h for −3, −2 and −1 days (6 p.m. +/− 1 h). Then, cats in the meloxicam group were treated subcutaneously with meloxicam at a dosage of 0.3 mg/kg body weight (equivalent to 0.1 mL/kg; Metacam^®^ injectable, Boehringer Ingelheim Vetmedica, inc) every 24 h up to 31 days. Meloxicam at 0.3 mg/kg body weight corresponds to the dose approved by the Food and Drug Administration. Cats in the control group received a 0.1 mL/kg body weight of saline subcutaneously every 24 h. At the end of the treatment phase, 3 cats in each group were euthanized within 48 h of the last treatment. The remaining cats in each group were euthanized 2 weeks after stopping the administration of the treatments. Intravenous overdose of pentobarbital (Beuthanasia-D, Intervet/Merck Animal Health, Giralda Farms, Madison, NJ, USA) was used for animal termination.

### Blood and urine sampling procedures

Blood (1.2 mL) and urine (0.5–4.0 mL) samples were collected before the first administration (0 h) and at 4, 9, 13, 17, 23, 26, 31, 34, 40 and 47 days after starting the administration of the treatments. Food was withheld 8 h prior to collection of blood and urine samples. Blood samples were collected from a jugular VAPs into Vacutainer^®^ tubes (Becton-Dickinson, inc) containing EDTA during the evening (6 p.m. +/− 1 h) prior to administration of treatments. Urine samples were collected into 15 mL conical centrifuge tubes immediately after collection of blood samples by ultrasound-guided cystocentesis following standard procedures. Immediately upon collection, blood and urine samples were placed on ice. Within 15 min of each sample collection, blood and urine samples were centrifuged at 1800 × *g* for 8 min. Supernatant (200 uL) of each sample was aliquoted into cryotubes and stored at −80 °C until analysis.

### Determination of lipids in plasma and urine

The lipidome was determined in urine and plasma samples collected before the administration of the first treatment (time 1, baseline), and at 4 (time 2), 9 (time 3) and 13 (time 4) and 17 (time 5) days after the first administration of meloxicam. We evaluated the lipidome up to 17 days after the first administration of meloxicam because we were interested only in early plasma and urine lipidome changes. The definition of this sampling period was based on abnormal changes in serum creatinine and BUN concentrations in the meloxicam-treated cats. Lipidomic analyses were performed at the West Coast Metabolomics Center (Davis, CA, USA), a National Institute of Health Regional resource core. The extraction^40^ and chromatographic^41^ methods used to determine feline plasma and urine lipids are reported in Supplementary Method S1. Concentrations of the internal standards spiked in plasma and urine samples can be found in Supplementary Table S7. Lipids were classified according to the Lipid MAPS Consortium^42^ (Supplementary Table S1). Lipids were identified based on their retention time, mass spectral information and the Lipidblast library^43^. Lipids were reported based on database identifiers for Lipidblast^43^ and InChIKey (Supplementary Table S1).

### Serum BUN and creatinine determination

Frozen serum samples were submitted to IDEXX Laboratories (transported overnight on dry ice) within 48 h following collection for determination of serum BUN and creatinine.

### Statistical analysis

**Figure 5:**
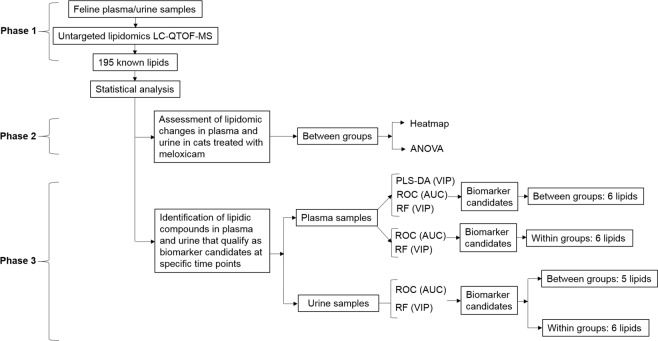
Experimental workflow to identify biomarker candidates for discriminating meloxicam and saline-treated cats. The workflow was applied to all the time points evaluated: pre-treatment (time 1), urine and plasma samples collected before the administration of the first treatment, and at 4 (time 2), 9 (time 3) and 13 (time 4) and 17 (time 5) days after the first administration of meloxicam.

The data were analyzed using a 3-phase approach as shown in Fig. 5. The data were analyzed by comparing the lipidome between the meloxicam and saline groups. In addition, we assessed the lipidome changes in the meloxicam-treated cats by comparing the lipidome at each sampling time after the administration of meloxicam against the baseline lipidome of the same group.

### Data analysis approach

As it is recommended, the peak intensities of each detected lipid were log (base 2) transformed to stabilize the variance before the comparison of the lipidome. In addition, the data was auto scaled (mean-centered and divided by the standard deviation of each variable^44^). All the analyses done in this study were performed using Metaboanalyst 4.0^45^, an open source R-based program specifically designed for metabolomics statistical analysis^46,47^.

### Differential analysis

Two-factor independent samples: two-way (between subjects) analysis of variance (ANOVA) was used to compare the data from the control and meloxicam groups over time. Benjamini-Hochberg false discovery rate method was used for multiple testing correction^48^. Heatmaps of significantly different lipids were generated to cluster lipids and a *p* < 0.05 was considered significant. The heatmaps were created using Euclidean distance and the Ward method of hierarchical clustering.

### Supervised dimension reduction

Partial least square-discriminatory analysis (PLS-DA) was used to determine variation between datasets for both, between and within groups, and at each time point. The predicting and fitting performance was assessed using the values of Q^2^ and R^2^, respectively^49^. R^2^ measures the goodness of fit while Q^2^ measures the predictive ability of the model^50^. The model was internally validated based on leave-one-out cross validation^51^. Variable importance in projection (VIP) value was used to select the most representative biomarker candidates. A lipid metabolite is considered important for the model when its VIP value is >1.0^33,52^.

### Random forest classification

Random forest was applied to classify lipids according to their contributions to the phenotype classification accuracy^35,53^. The parameters used for the analysis included 500 trees, 7 predictors and randomness. The acceptance criteria to consider a lipid as a putative biomarker candidate at this step was a value of mean decrease accuracy ≥0.004^54^.

### Receiving operating characteristic curve analysis

Receiving operating curves were used to evaluate the diagnostic performance of biomarker candidates. Receiving operating characteristic curves were summarized using the area under the curve (AUC) as a measure of how well a parameter can distinguish between cats in the control and meloxicam groups. AUC values of ∼0.5 and 1 indicate random and perfect performance, respectively. In this study, only those lipids with an AUC ROC > 0.85 were selected as biomarker candidates^34^.

## Supplementary information

Table S1

Supplementary Material

## Data Availability

The data generated during the current study are available from the corresponding author on reasonable request.
