# Supplementary material for: Repeated administration of the NSAID meloxicam alters the plasma and urine lipidome
Source: Sci Rep. 2019 Mar 13;9:4303. doi: 10.1038/s41598-019-40686-4 (PMC6416286; doi:10.1038/s41598-019-40686-4)
Supplement: Supplementary file 2 — Supplementary Material [file 41598_2019_40686_MOESM2_ESM.docx]

Supplementary Material - Repeated administration of the NSAID meloxicam alters the plasma and urine lipidome

Sol M. Rivera-Velez^1^, Liam E. Broughton-Neiswanger^1^, Martin Suarez^2^, Pablo Piñeyro^3^, Jinna Navas^1^, Sandy Chen^2^, Julianne Hwang^1^ & Nicolas F. Villarino^1,*^

1 Program in Individualized Medicine, Department of Veterinary Clinical Sciences, College of Veterinary Medicine, Washington State University, Pullman 99164, WA, United States.

2 Department of Veterinary Clinical Sciences, College of Veterinary Medicine, Washington State University, Pullman 99164, WA, United States.

3 Veterinary Diagnostic Laboratory, College of Veterinary Medicine, Iowa State University, Ames 1134, IA, United States.

* [nicolas.villarino@wsu.edu](mailto:nicolas.villarino@wsu.edu)

**Table 2.** List of lipids whose signal intensities were statistically different (*p* < 0.05) in plasma from cats treated with saline or meloxicam at 0.3 mg/kg every 24 h for up to 31 days based on ANOVA test. The *p* values reported correspond to adjusted *p* values based on the false discovery rate criteria.

| Name | phenotype(adj.p) |  | Name | phenotype(adj.p) |
| --- | --- | --- | --- | --- |
| Acylcarnitine C18:0 | 0.016 |  | TG (46:3) | 0.008 |
| CE (16:1) | 0.048 |  | TG (48:1) | ˂0.001 |
| CE (18:2) | 0.005 |  | TG (48:2) | 0.012 |
| CE (20:4) | 0.003 |  | TG (48:3) | 0.024 |
| CE (22:6) | ˂0.001 |  | TG (49:1) | ˂0.001 |
| DG (36:3) | ˂0.001 |  | TG (49:2) | 0.002 |
| DG (36:4) | ˂0.001 |  | TG (50:1) | ˂0.001 |
| DG (38:5) | 0.005 |  | TG (50:2) | ˂0.001 |
| DG (38:6) | 0.003 |  | TG (50:3) | 0.002 |
| LPC (18:0) | 0.005 |  | TG (50:4) | 0.006 |
| LPC (20:0) | 0.015 |  | TG (51:1) | ˂0.001 |
| PC (16:0/9:0(CHO)) | 0.030 |  | TG (51:2) | ˂0.001 |
| PC (34:1) | 0.002 |  | TG (51:3) | ˂0.001 |
| PC (34:2) | 0.002 |  | TG (51:4) | 0.001 |
| PC (36:1) | 0.002 |  | TG (52:1) | ˂0.001 |
| PC (36:2) | 0.001 |  | TG (52:2) | ˂0.001 |
| PC (36:4)A | 0.016 |  | TG (52:3) | ˂0.001 |
| PC (36:4)B | 0.002 |  | TG (52:4) | ˂0.001 |
| PC (37:6) | 0.038 |  | TG (52:5) | ˂0.001 |
| PC (38:3) | ˂0.001 |  | TG (52:6) | 0.019 |
| PC (38:4)A | 0.016 |  | TG (53:1) | ˂0.001 |
| PC (38:6)A | ˂0.001 |  | TG (53:3) | ˂0.001 |
| PC (38:6)C | 0.028 |  | TG (53:4) | ˂0.001 |
| PC (40:4) | ˂0.001 |  | TG (53:5) | ˂0.001 |
| PC (40:5)A | 0.005 |  | TG (54:1) | ˂0.001 |
| PC (40:5)B | ˂0.001 |  | TG (54:3) | ˂0.001 |
| PC (40:6)B | ˂0.001 |  | TG (54:4) | ˂0.001 |
| PC (40:8) | 0.007 |  | TG (54:5)A | ˂0.001 |
| PC (p-40:5) | 0.003 |  | TG (54:5)B | ˂0.001 |
| PE (34:2) | ˂0.001 |  | TG (54:6)A | ˂0.001 |
| PE (38:4) | ˂0.001 |  | TG (54:6)B | ˂0.001 |
| PE (p-36:2) | 0.005 |  | TG (56:3) | ˂0.001 |
| PE (p-36:4) | 0.006 |  | TG (56:1) | 0.010 |
| SM (d34:1) | 0.033 |  | TG (56:2) | ˂0.001 |
| SM (d34:2) | ˂0.001 |  | TG (56:4) | ˂0.001 |
| SM (d36:0) | 0.002 |  | TG (56:6) | ˂0.001 |
| SM (d36:1) | ˂0.001 |  | TG (56:7) | ˂0.001 |
| SM (d36:2) | ˂0.001 |  | TG (56:8) | 0.001 |
| SM (d37:1) | 0.007 |  | TG (58:1) | 0.050 |
| SM (d38:1) | 0.002 |  | TG (58:2) | 0.008 |
| SM (d38:2) | ˂0.001 |  | TG (58:3) | 0.027 |
| SM (d40:2)A | 0.018 |  | TG (58:6) | ˂0.001 |
| SM (d40:2)B | 0.014 |  | TG (58:8) | 0.001 |
| SM (d42:2)A | 0.012 |  | TG (16:0/18:2/18:3) | ˂0.001 |
| TG (46:0) | 0.038 |  | TG (16:1/16:1/18:2) | 0.016 |
| TG (46:1) | 0.014 |  | TG (53:2) | ˂0.001 |
| TG (46:2) | 0.008 |  | TG (54:2) | ˂0.001 |

**Table 3.** List of lipids in plasma from cats treated with saline (n=6) or meloxicam (n=6) at 0.3 mg/kg every 24 h for up to 31 days based on variable importance in projection (VIP > 1) values generated by partial least square-discriminatory analysis (PLS-DA) for plasma samples collected at different time points. The values of VIP for the lipids at time 3 are not described in this table because the PLS-DA model did not fit the data.

| Time 2 |  | Time 4 |  | Time 5 |  |
| --- | --- | --- | --- | --- | --- |
| Name | **VIP value** | **Name** | **VIP value** | **Name** | **VIP value** |
| Acylcarnitine C16:0 | 1.146 | CE (16:1) | 1.081 | Acylcarnitine C16:0 | 1.133 |
| Acylcarnitine C18:0 | 1.380 | CE (22:6) | 1.010 | Acylcarnitine C18:0 | 1.420 |
| CE (20:4) | 1.187 | DG (36:4) | 1.424 | Acylcarnitine C18:1 | 1.294 |
| CE (22:6) | 1.676 | LPC (14:0) | 1.646 | CE (20:4) | 1.355 |
| DG (36:4) | 1.670 | LPC (15:0) | 1.484 | CE (22:6) | 1.379 |
| LPC (14:0) | 1.102 | LPC (16:1) | 1.190 | LPC (22:6) | 1.065 |
| LPC (15:0) | 1.208 | LPC (17:1) | 1.168 | PC (40:5)A | 1.323 |
| LPC (16:1) | 1.159 | LPC (18:3) | 1.551 | PC (40:6)B | 1.539 |
| LPC (18:3) | 1.648 | LPC (20:0) | 1.667 | PC (40:7) | 1.120 |
| LPC (20:1) | 1.048 | PC (31:0) | 1.408 | PC (p-38:2) | 1.234 |
| PC (40:5)A | 1.022 | PC (32:1) | 1.022 | PC (p-40:5) | 1.259 |
| PC (40:6)B | 1.113 | PC (32:2) | 1.353 | SM( d34:1) | 1.035 |
| PC (p-40:5) | 1.235 | PC (33:0) | 1.092 | SM (d36:0) | 1.342 |
| PC (p-40:6)B | 1.441 | PC( 3:4)3 | 1.574 | SM (d37:1) | 1.285 |
| PC (p-4:25) | 1.371 | PC (34:4) | 1.364 | SM (d42:2)A | 1.323 |
| PE (p-36:4) | 1.387 | PC (p-34:1)A | 1.169 | TG (46:0) | 1.245 |
| SM (d36:0) | 1.266 | PC (p-34:2) | 1.021 | TG (48:1) | 1.207 |
| SM (d37:1) | 1.225 | PC (p-36:2) | 1.199 | TG (49:0) | 1.433 |
| SM (d41:1) | 1.108 | PC (p-38:5) | 1.264 | TG (49:1) | 1.568 |
| SM (d41:2) | 1.104 | PC (p-40:3) | 1.350 | TG (49:2) | 1.126 |
| SM (d42:2)A | 1.027 | SM (d32:0) | 1.355 | TG (50:0) | 1.379 |
| TG (16:0/18:2/18:3) | 1.463 | SM (d32:1) | 1.016 | TG (51:1) | 1.854 |
| TG (16:1/16:1/18:2) | 1.104 | SM (d39:1) | 1.165 | TG (54:1) | 1.772 |
| TG (46:3) | 1.200 | TG (46:1) | 1.612 | TG (56:1) | 1.345 |
| TG (48:1) | 1.004 | TG (46:2) | 1.757 | TG (56:2) | 1.473 |
| TG (49:1) | 1.192 | TG (48:1) | 1.578 | TG (56:3) | 1.538 |
| TG (50:0) | 1.487 | TG (48:2) | 1.381 | TG (56:8) | 1.154 |
| TG (51:1) | 1.311 | TG (48:3) | 1.179 | TG (58:1) | 1.307 |
| TG (52:6) | 1.142 | TG (49:1) | 1.720 | TG (58:2) | 1.490 |
| TG (54:1) | 1.425 | TG (49:2) | 1.430 | TG (58:6) | 1.521 |
| TG (56:3) | 1.054 | TG (51:1) | 1.756 | TG (60:2) | 1.088 |
| TG (56:8) | 1.041 | TG (54:1) | 1.779 |  |  |
| TG (58:6) | 1.159 | TG (56:1) | 1.166 |  |  |
|  |  | TG (56:3) | 1.306 |  |  |
|  |  | TG (56:8) | 1.377 |  |  |
|  |  | TG (58:1) | 1.195 |  |  |
|  |  | TG (58:2) | 1.022 |  |  |
|  |  | TG (58:6) | 1.312 |  |  |
|  |  | TG (60:2) | 1.057 |  |  |
|  |  | TG (16:1/16:1/18:2 | 1.245 |  |  |

**Table 4.** List of lipids in plasma that could be used to discriminate meloxicam- and saline-treated cats at different time points (upper panel). The lower panel of the table lists the lipids whose signal intensity changed from baseline and could be considered as biomarker candidates to monitor over time the effect of meloxicam on the lipidome. The list of lipids was generated using the lipidome data at each sample time from saline-treated (n=6) or meloxicam-treated (n=6) cats at 0.3 mg/kg every 24 h for up to 31 days. All the listed lipids met the pre-defined acceptance criteria; (i) an AUC ROC > 0.85, (ii) a VIP ≥ 1 (Table S3), (iii) a value of mean decrease accuracy ≥ 0.004 (Fig. S4 and S5) and (iv) a *p* value ˂ 0.05. The intensity fold change for each lipid was calculated based on the intensity mean ratios of each group (meloxicam-treated (n=6) / saline-treated (n=6) cats).

| Plasma metabolites | Chemical family | Source | AUC ROC | *p* value | Fold change | Time |
| --- | --- | --- | --- | --- | --- | --- |
| Between meloxicam and control groups | | | | | | |
| DG (36:4) | Glycerolipids | Endogenous and food | 1.000 | ˂0.001 | 2.200 | 2 |
| TG (56:2) | Glycerolipids | Endogenous and food | 0.920 | 0.030 | 3.320 | 3 |
| TG (54:1) | Glycerolipids | Endogenous and food | 1.000 | 0.003 | 2.486 | 4 |
| TG (49:1) | Glycerolipids | Unknown | 0.972 | 0.004 | 2.089 | 4 |
| DG (36:4) | Glycerolipids | Endogenous and food | 0.944 | 0.029 | 2.994 | 4 |
| TG (51:1) | Glycerolipids | Unknown | 1.000 | ˂0.001 | 4.074 | 5 |
| TG (54:1) | Glycerolipids | Endogenous and food | 1.000 | ˂0.001 | 2.7824 | 5 |
| TG (56:3) | Glycerolipids | Endogenous and food | 0.944 | 0.006 | 1.923 | 5 |
| TG (56:2) | Glycerolipids | Endogenous and food | 0.889 | 0.010 | 1.639 | 5 |
| Within meloxicam group (against baseline) | | | | | | |
| TG (51:1) | Glycerolipids | Unknown | 0.972 | 0.006 | 2.535 | 5 |
| TG (49:1) | Glycerolipids | Unknown | 0.917 | 0.004 | 2.286 | 5 |
| TG (56:6) | Glycerolipids | Endogenous and food | 0.944 | 0.003 | 1.844 | 5 |
| TG (48:1) | Glycerolipids | Endogenous and food | 0.889 | 0.021 | 2.187 | 5 |
| TG (54:6)B | Glycerolipids | Endogenous and food | 0.944 | 0.013 | 2.033 | 5 |
| SM (d42:2)A | Sphingolipids | Unknown | 0.861 | 0.013 | 1.855 | 5 |

**Table 5.** List of lipids in urine that could be used to discriminate meloxicam- and saline-treated cats at different time points (upper panel). The lower panel of the table lists the lipids whose signal intensity changed from baseline and could be considered as biomarker candidates to monitor over time the effect of meloxicam on the lipidome. The list of lipids was generated using the lipidome information at each sample time from saline-treated (n=6) or meloxicam-treated (n=6) cats at 0.3 mg/kg every 24 h for up to 31 days. All the listed lipids met the pre-defined acceptance criteria; (i) an AUC ROC > 0.85, (ii) a value of mean decrease accuracy ≥ 0.004 (Fig. S6 and S7) and (iii) a *p* value ˂ 0.05. The intensity fold change for each lipid was calculated based on the intensity mean ratios of each group (meloxicam-treated (n=6) / saline-treated (n=6) cats).

| Urine metabolites | Chemical family | Source | AUC  ROC | *p* value | Fold change | Time |
| --- | --- | --- | --- | --- | --- | --- |
| Between meloxicam and control groups | | | | | | |
| LPC (16:1) | Glycerophospholipids | Endogenous and food | 0.972 | 0.005 | 0.500 | 2 |
| PC (33:1) | Glycerophospholipids | Unknown | 0.917 | 0.014 | 0.620 | 3 |
| LPC (16:1) | Glycerophospholipids | Unknown | 1.000 | 0.008 | 0.477 | 3 |
| PC (35:4)B | Glycerophospholipids | Unknown | 1.000 | 0.034 | 1.995 | 3 |
| PC (36:5)B | Glycerophospholipids | Unknown | 0.972 | 0.041 | 1.975 | 3 |
| PC (36:6) | Glycerophospholipids | Endogenous and food | 0.944 | 0.008 | 3.396 | 5 |
| Within meloxicam group (against baseline) | | | | | | |
| LPC (17:1) | Glycerophospholipids |  | 1.000 | 0.001 | 0.232 | 2 |
| Acylcarnitine (C16:0) | Fatty acyls | Endogenous and food | 1.000 | 0.013 | 1.567 | 2 |
| LPC (16:1) | Glycerophospholipids | Endogenous and food | 0.861 | 0.048 | 0.577 | 2 |
| LPC (17:1) | Glycerophospholipids | Unknown | 0.917 | 0.030 | 0.589 | 3 |
| LPC (16:1) | Glycerophospholipids | Unknown | 0.889 | 0.026 | 0.481 | 3 |
| PC (32:1) | Glycerophospholipids | Endogenous and food | 0.917 | 0.003 | 0.607 | 3 |
| PC (p-36:3) | Glycerophospholipids | Endogenous and food | 1.000 | ˂0.001 | 7.424 | 5 |
| PE (p-36:4) | Glycerophospholipids | Endogenous and food | 0.944 | 0.006 | 0.461 | 5 |

**Table 6.** Concentration of lipids in plasma and urine that were identified as biomarker candidates for discriminating meloxicam-treated (Meloxicam) and saline-treated (Control) cats (comparison between groups); and baseline (BM) and meloxicam group (M) (comparison within groups) at different times after the administration of saline or meloxicam at 0.3 mg/kg every 24 h for up to 17 days (times 2 to 5). Potential biomarker candidates were quantified based on responses of internal standards.

| **Time** | **Matrix** | **Annotation** | **Control conc. ng/mL (x̄ ± SD)** | **SD** | **Meloxicam conc. ng/mL (x̄ ± SD)** | **SD** | **Comparison** |
| --- | --- | --- | --- | --- | --- | --- | --- |
| 2 | Plasma | DG (36:4) | 404 | 152 | 833 | 180 | Between groups |
| 3 | Plasma | TG (56:2) | 245 | 241 | 611 | 791 |  |
| 4 | Plasma | DG (36:4) | 397 | 95 | 922 | 373 |  |
| 4 | Plasma | TG (49:1) | 230 | 52 | 640 | 355 |  |
| 4 | Plasma | TG (54:1) | 169 | 38 | 559 | 373 |  |
| 5 | Plasma | TG (51:1) | 287 | 244 | 1032 | 470 |  |
| 5 | Plasma | TG (54:1) | 270 | 192 | 677 | 191 |  |
| 5 | Plasma | TG (56:2) | 189 | 108 | 288 | 99 |  |
| 5 | Plasma | TG (56:3) | 323 | 164 | 618 | 330 |  |
| 2 | Urine | LPC (16:1) | 42 | 16 | 21 | 6.8 |  |
| 3 | Urine | LPC (16:1) | 40 | 17 | 16 | 6.9 |  |
| 3 | Urine | PC (33:1) | 25 | 8.2 | 15 | 3.4 |  |
| 3 | Urine | PC (35:4)B | 9.0 | 1.1 | 12 | 1.0 |  |
| 3 | Urine | PC (36:5)B | 9.1 | 1.4 | 12 | 2.4 |  |
| 5 | Urine | PC (36:6) | 11 | 6.3 | 18 | 5.7 |  |
| **Time** | **Matrix** | **Annotation** | **BM conc. ng/mL (x̄ ± SD)** | **SD** | **Meloxicam conc. ng/mL (x̄ ± SD)** | **SD** | **Comparison** |
| 5 | Plasma | SM (d42:2)A | 5024 | 553 | 7929 | 2413 | Within groups |
| 5 | Plasma | TG (48:1) | 1647 | 579 | 3318 | 1943 |  |
| 5 | Plasma | TG (49:1) | 515 | 129 | 1059 | 470 |  |
| 5 | Plasma | TG (51:1) | 445 | 210 | 1032 | 470 |  |
| 5 | Plasma | TG (54:6)B | 1589 | 654 | 2928 | 1756 |  |
| 5 | Plasma | TG (56:6) | 4353 | 1420 | 7106 | 3004 |  |
| 2 | Urine | LPC (16:1) | 36 | 15 | 21 | 6.8 |  |
| 2 | Urine | LPC (17:1) | 15 | 4.6 | 6.7 | 1.0 |  |
| 2 | Urine | AC (16:0)* | 17 | 4.2 | 26 | 6.4 |  |
| 3 | Urine | LPC (16:1) | 36 | 15 | 16 | 6.9 |  |
| 3 | Urine | LPC (17:1) | 15 | 4.6 | 8.3 | 3.9 |  |
| 3 | Urine | PC (32:1) | 77 | 17 | 47 | 7.3 |  |
| 5 | Urine | PC (p-36:3) | 37 | 13 | 136 | 38 |  |
| 5 | Urine | PE (p-36:4) | 610 | 234 | 251 | 90 |  |

*AC, acylcarnitine

**Table 7.** Concentrations of the internal standards spiked in plasma and urine samples.

| **Internal Standard** | **µg/mL plasma** | **µg/mL urine** |
| --- | --- | --- |
| 22:1 Cholesterol ester | 1455.6 | 494.9 |
| Cer(d18:1/17:0) | 10.6 | 3.6 |
| d_7_-Cholesterol | 42.4 | 14.4 |
| DG(12:0/12:0/0:0) | 42.4 | 14.4 |
| DG(18:1/2:0/0:0) | 254.5 | 86.5 |
| LPC(17:0) | 21.2 | 7.2 |
| LPE(17:1) | 10.6 | 3.6 |
| MG(17:0/0:0/0:0) | 84.8 | 28.8 |
| PC(12:0/13:0) | 0.8 | 0.3 |
| PE(17:0/17:0) | 31.8 | 10.8 |
| SM 17:0 (d18:1/17:0) | 8.5 | 2.9 |
| Sphingosine (d17:1) | 4.7 | 1.6 |
| d_5_-TG(17:0/17:1/17:0) | 13.3 | 4.5 |

**Figure 1.** Serum creatinine concentration (mean + SD) *vs.* time profile in saline-treated (n=6) and meloxicam-treated (n=6) cats treated at 0.3 mg/kg every 24 h for up to 31 days. After 31 days of treatment, 3 cats in each group were euthanized. The remaining cats (3 in each group) were monitored for another 2 weeks. The concentration of creatinine was assessed is serum samples from cats collected during the pre-induction, induction and post-induction stages of the study. The median creatinine serum concentration in the meloxicam group was not different than that of the control group (p > 0.05) at any time point. Before the administration of the treatments, all cats had comparable serum concentration levels of creatinine. All cats had serum creatinine levels < 1.7 mg/dL (normal upper range). The mean (±SD) creatinine concentration (mg/dL) was 0.94 (0.13) and 0.88 (0.13) and 0.9 (0.13) and 0.9 (0.07) at -4 and 0 h, for the control and meloxicam groups, respectively. In the control group during the induction phase, the serum concentration of creatinine remained relatively steady during the duration of this phase. There were not statistical differences between the mean concentration of creatinine at the baseline and last sampling time of the induction phase. In the meloxicam group, the mean serum creatinine concentration was comparable to that observed in the control group up to 9 days following the first administration of meloxicam. Afterward, there was a noticeable increment of the mean serum concentration of creatinine (range 5-42 %). After stopping the administration of the treatments (post-induction phase), the serum concentration of creatinine was monitored in 3 out of 6 cats for each treatment group (C_1, C_2, and C_3 for the control group and M_2, M_3 and M_4 for the meloxicam group). The remaining cats were terminated within 24 h after the last treatment. The serum concentration of creatinine in the control group remained relatively constant and below 1.7 mg/mL during the duration of this phase. In the meloxicam group, the mean serum concentration of creatinine tended to decrease after stopping the administration of meloxicam.

**Figure 2.** BUN concentration (mean + SD) *vs.* time profile in saline-treated (n=6) and meloxicam-treated (n=6) cats treated at 0.3 mg/kg every 24 h for up to 31 days. After 31 days of treatment, 3 cats in each group were euthanized. The remaining cats (3 in each group) were monitored for another 2 weeks. The concentration of BUN was assessed in serum samples from cats collected during the pre-induction, induction and post-induction stages of the study. Before the administration of the treatments (pre-induction phase), all the cats had comparable serum concentration levels of BUN. The mean (±SD) serum BUN concentration was 24.6 (1.86) and 24.8 (2.22) and 25 (2.09) and 23.5 mg/dL (1.87) at -4 and 0 h, for the control and meloxicam groups, respectively. During the induction phase in the control group, the serum concentration of BUN remained relatively steady during the duration of this phase. There were not statistical differences between the mean concentration of creatinine at the baseline and last sampling time of the induction phase (p > 0.05). In this group, the maximum increment in the serum concentration was < 60%. In the meloxicam group, the BUN serum concentration increased markedly in 3 out 6 cats. At the end of this phase, 3 out of 6 cats had BUN serum concentration comparable to the baseline levels while the 3 remaining cats had BUN serum concentration at least 2-fold higher than their corresponding baseline levels. After stopping the administration of the treatments, the serum concentration of BUN in the control cats (C_1, C_2, and C_3) remained relatively constant and below 35 mg/mL during the duration of this phase. In the meloxicam group, the mean serum concentration of BUN tended to decrease after stopping the administration of meloxicam. At the termination time point, the BUN serum concentrations were 27, 29 and 51 mg/dL for cats M_2, M_4, and M_3, respectively.

**
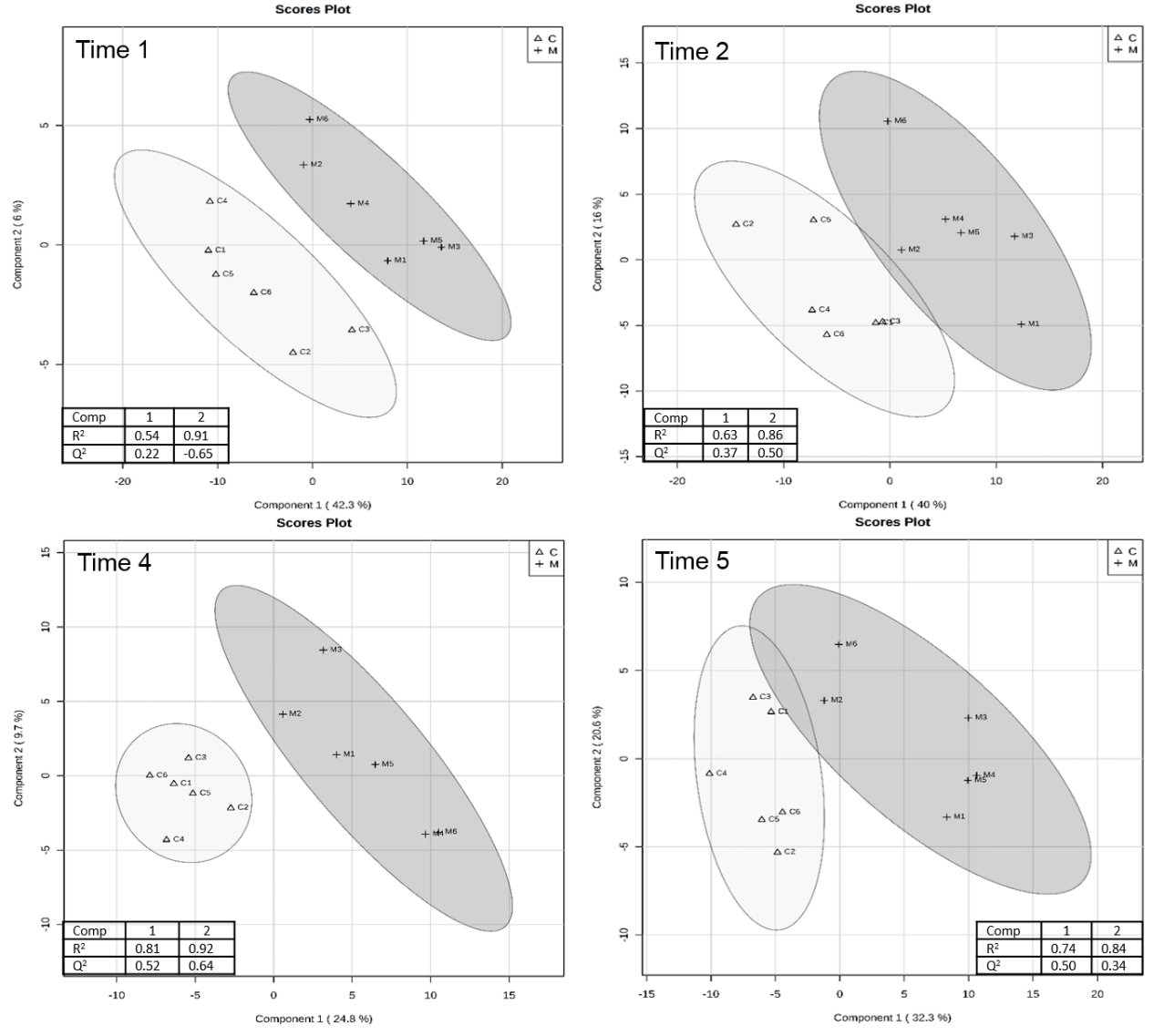
**

**Figure 3.** Score plots of the PLS-DA model for the plasma lipidome obtained at sampling times 1, 2, 4 and 5 from saline-treated (n=6) or meloxicam-treated (n=6) cats at 0.3 mg/Kg every 24 h for up to 31 days. C: saline-treated cats. M: meloxicam-treated cats. Each data point represents an individual plasma sample. Points that are close together represent samples with similar metabolic phenotypes; points that are far away have dissimilar metabolic phenotypes. Shaded backgrounds define the 95% confidence interval for each group. The R^2^ and Q^2^ were ≥ 0.5 for times 2, 4 and 5 for the component 1 and/or 2, except for times 1 and 3.


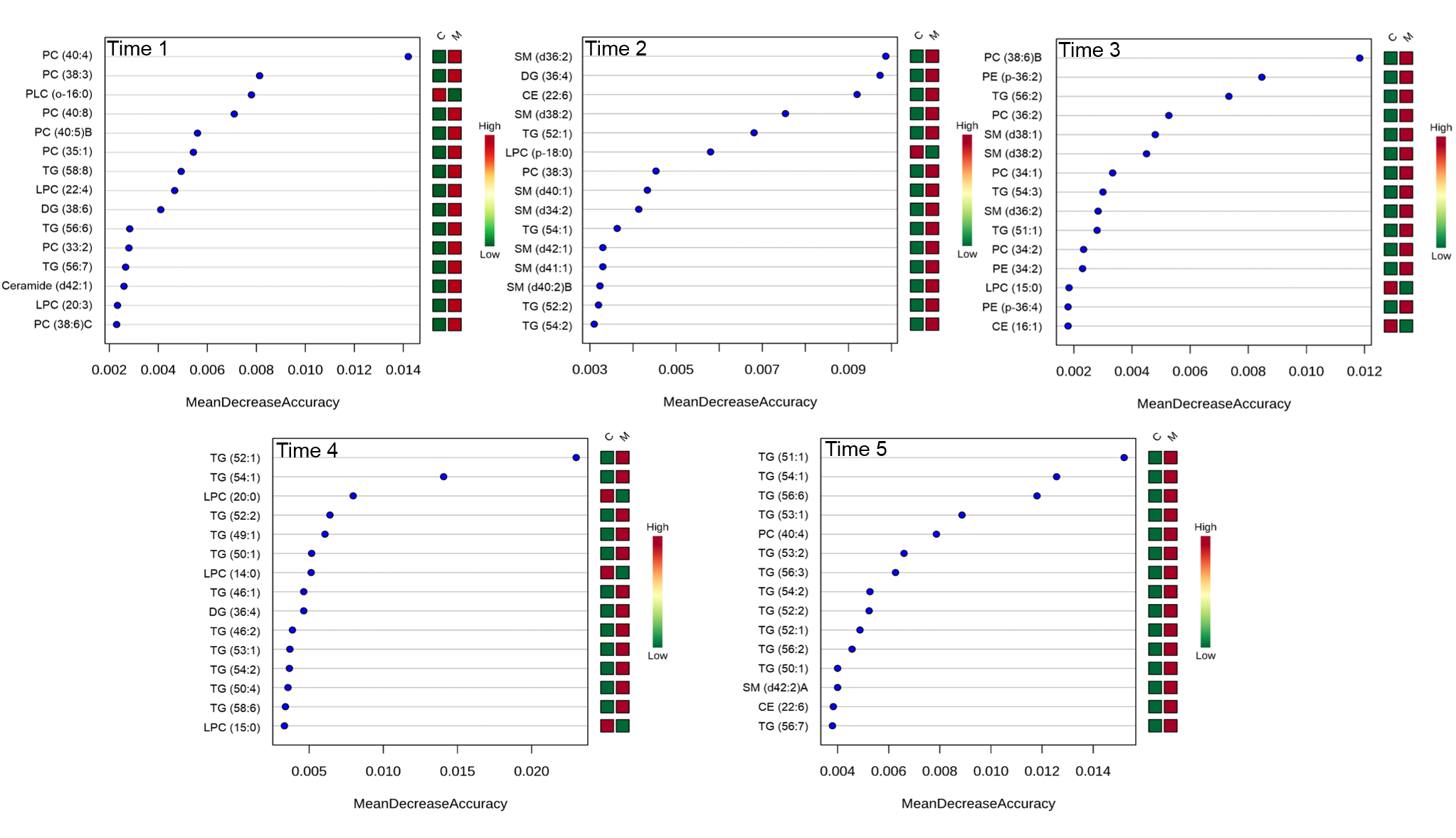


**Figure 4.** Random forest variable importance plots for plasma samples for identifying lipids that discriminate meloxicam-treated (n=6) and saline-treated (n=6) cats at each sampling time (2-5) after the administration of the treatments. Lipids with the mean decrease accuracy ≥ 0.004 were considered relevant for the predictive model.


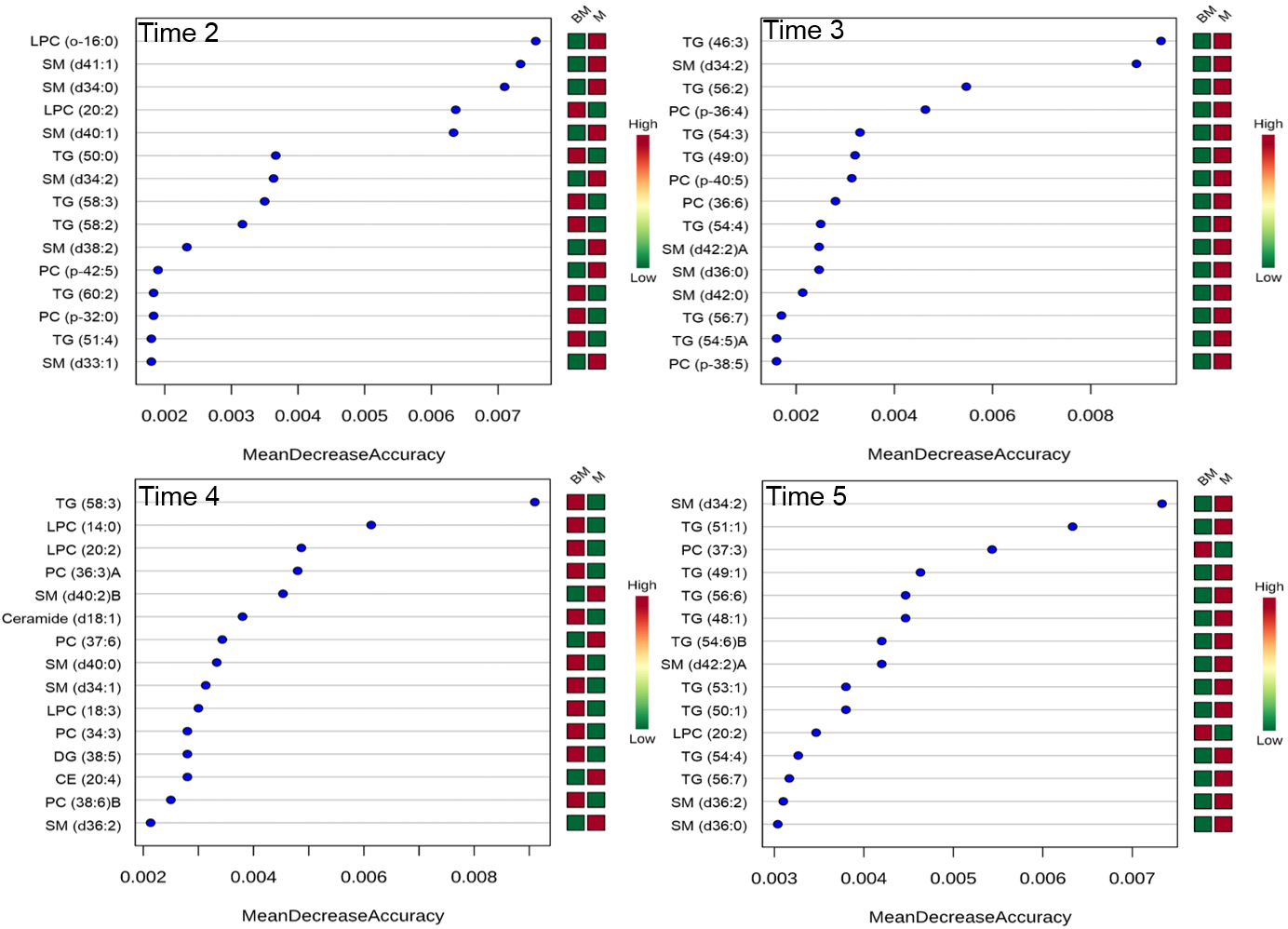


**Figure 5.** Random forest variable importance plots for plasma samples for identifying lipids that discriminate meloxicam-treated cats before the administration of meloxicam (BM) (n=6) and at each sampling time after the administration of meloxicam (M) (n=6)**.** Lipids with the mean decrease accuracy ≥ 0.004 were considered relevant for the predictive model.


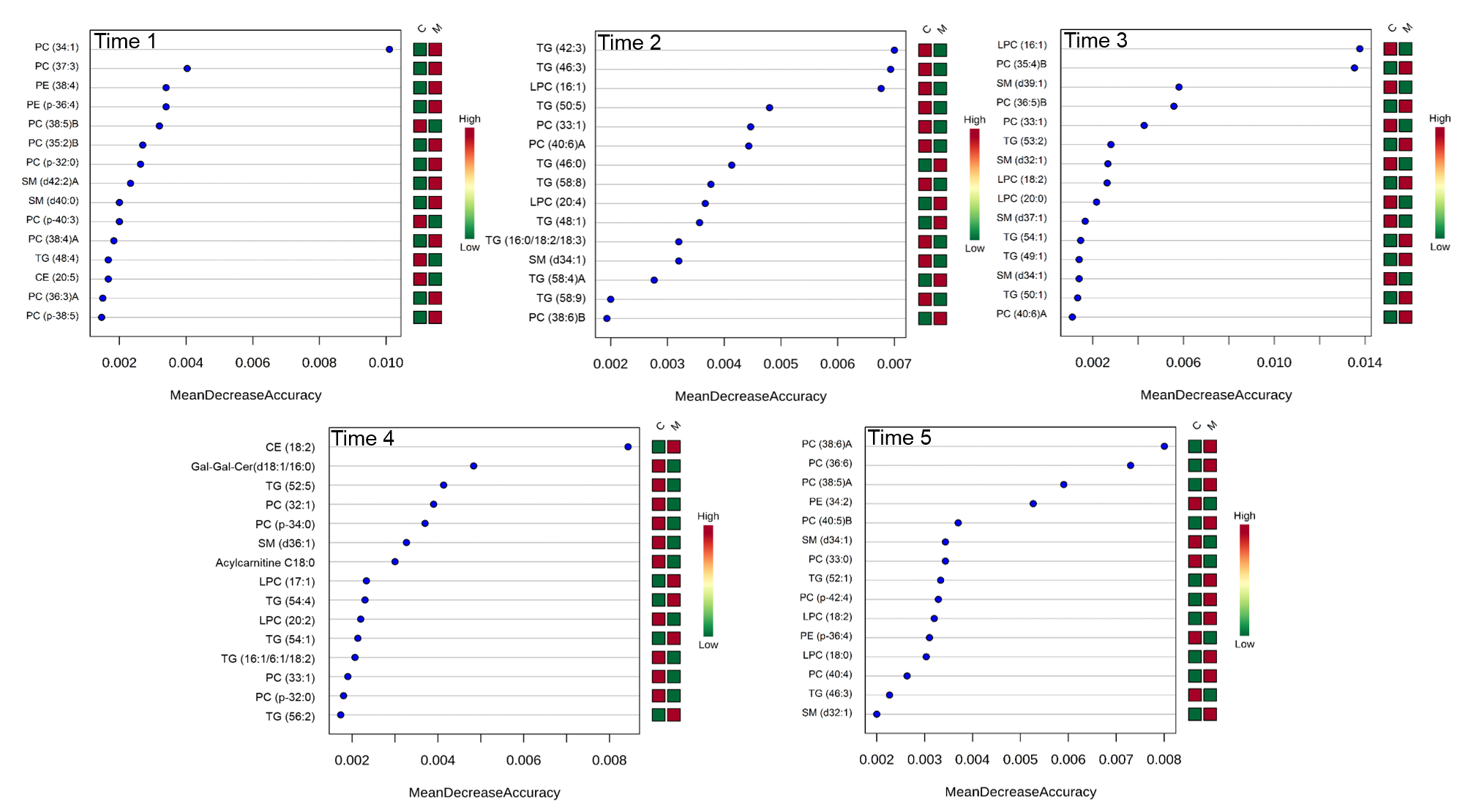


**Figure 6.** Random forest variable importance plots for urine samples for identifying lipids that discriminate meloxicam-treated (n=6) and saline-treated (n=6) cats at each sampling time (2-5) after the administration of the treatments. Lipids with the mean decrease accuracy ≥ 0.004 were considered relevant for the predictive model.


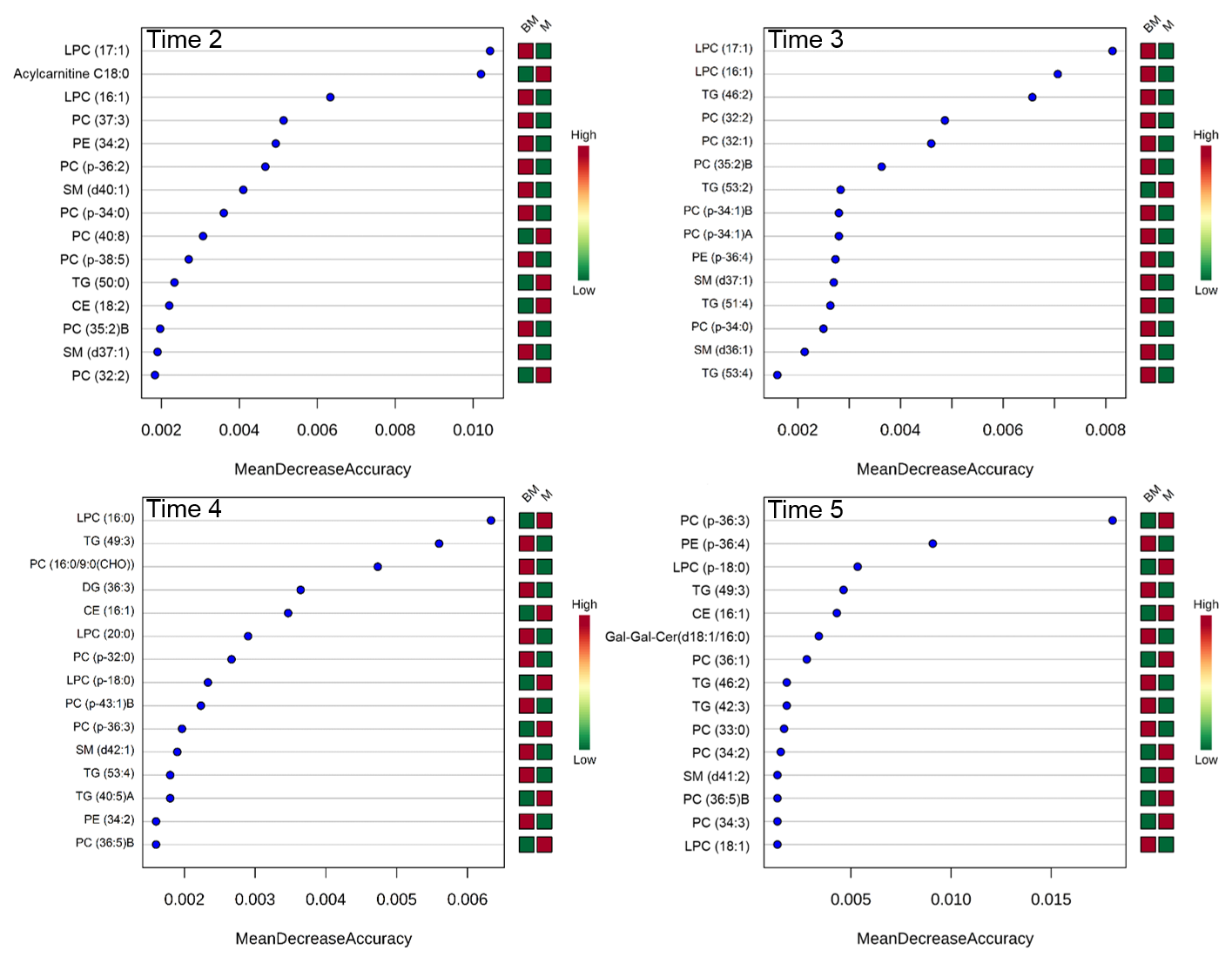


**Figure 7.** Random forest variable importance plots for urine samples for identifying lipids that discriminate meloxicam-treated cats before the administration of meloxicam (BM) (n=6) and at each sampling time after the administration of meloxicam (M) (n=6)**.** Lipids with the mean decrease accuracy ≥ 0.004 were considered relevant for the predictive model.


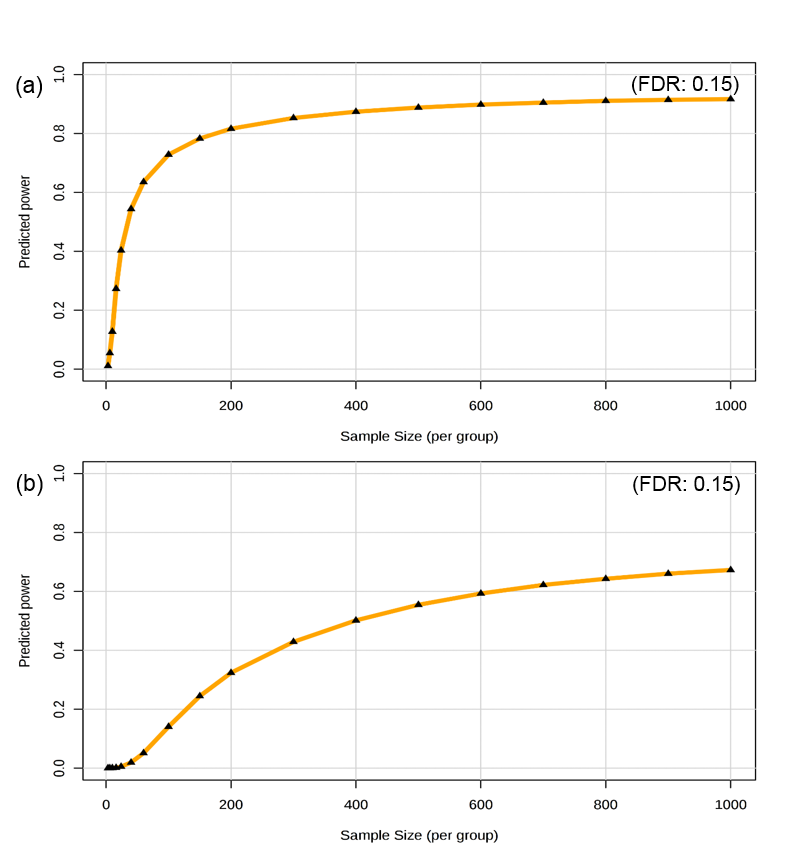


**Figure 8.** Multidimensional statistical power analysis of plasma (a) and urine (b) lipidome from meloxicam-treated (n=6) and saline-treated cats (n=6) as implemented by Metaboanalyst 4.0 (https://www.metaboanalyst.ca/). As it is recommended, the peak intensities of each detected lipid were log (base 2) transformed to stabilize the variance before the comparison of the lipidome. In addition, the data was auto scaled (mean-centered and divided by the standard deviation of each variable19). As it recommended for high-dimensional metabolomic data, false discovery rate (FDR) was used as significance criterion. The effect size was estimated from the data. Effect size is defined as the differences of two groups means divided by the pooled standard deviation. The power calculation is based on two assumptions; 1) the effect is indeed present in the data, and 2) the test statistic follows a normal or near normal (Student‘s distribution).

TG (56:2) Time 3

DG (36:4) Time 2

TG (54:1) Time 4

TG (49:1) Time 4

DG (36:4) Time 4

TG (51:1) Time 5

TG (56:3) Time 5

TG (54:1) Time 5

TG (56:2) Time 5

**Figure 9.** Unidimensional statistical power analysis of biomarker candidates identified in plasma for discriminating meloxicam-treated (n=6) from saline-treated cats (n=6) at different time points after the administration of the treatments. The effect size was estimated from the data. Effect size is defined as the differences of two group means for each particular lipid. The standard deviation (SD) considered for each calculation corresponded to the largest SD relative to the mean for each particular metabolite. It is important to remark the selection of the effect size for the power calculations is arbitrary because the magnitude of the effect of interest in the population (effect size) necessary to calculate the sample sizes is still unknown. The power calculation is based on two assumptions; 1) the effect is indeed present in the data, and 2) the test statistic follows a normal or near normal (Student‘s distribution) α=0.05 and 1-β=0.8.The power calculations were done using Minitab V.18, State College PA.

PC (33:1) Time 3

LPC (16:1) Time 2

LPC (16:1) Time 3

PC (35:4)B Time 3

PC (36:5)B Time 3

PC (36:6) Time 5

**Figure 10.** Unidimensional statistical power analysis of biomarker candidates identified in urine for discriminating meloxicam-treated (n=6) from saline-treated cats (n=6) at different time points after the administration of the treatments. The effect size was estimated from the data. Effect size is defined as the differences of two group means for each particular lipid. The standard deviation (SD) considered for each calculation corresponded to the largest SD relative to the mean for each particular metabolite. It is important to remark the selection of the effect size for the power calculations is arbitrary because the magnitude of the effect of interest in the population (effect size) necessary to calculate the sample sizes is still unknown. The power calculation is based on two assumptions; 1) the effect is indeed present in the data, and 2) the test statistic follows a normal or near normal (Student‘s distribution) α=0.05 and 1-β=0.8.The power calculations were done using Minitab V.18, State College PA.

TG (49:1) Time 5

TG (51:1) Time 5

TG (56:6) Time 5

TG (48:1) Time 5

TG (54:6)B Time 5

SM (d42:2)A

Time 5

**Figure 11.** Unidimensional statistical power analysis of biomarker candidates identified in plasma within the meloxicam treated group (against baseline). The effect size was estimated from the data. Effect size is defined as the differences of two group means for each particular lipid. The standard deviation (SD) considered for each calculation corresponded to the larges SD relative to the mean for each particular metabolite. It is important to remark the selection of the effect size for the power calculations is arbitrary because the magnitude of the effect of interest in the population (effect size) necessary to calculate the sample sizes is still unknown. The power calculation is based on two assumptions; 1) the effect is indeed present in the data, and 2) the test statistic follows a normal or near normal (Student‘s distribution) α=0.05 and 1-β=0.8.The power calculations were done using Minitab V.18, State College PA.

LPC(16:1) Time 3

Acylcarnitine C16

Time 2

LPC(17:1) Time 3

LPC(17:1) Time 2

PC (32:1) Time 3

LPC(16:1) Time 3

PC(p-36:3) Time 5

PE(p-36:4) Time 5

**Figure 12.** Unidimensional statistical power analysis of biomarker candidates identified in urine within the meloxicam treated group (against baseline). The effect size was estimated from the data. Effect size is defined as the differences of two group means for each particular lipid. The standard deviation (SD) considered for each calculation corresponded to the larges SD relative to the mean for each particular metabolite. It is important to remark the selection of the effect size for the power calculations is arbitrary because the magnitude of the effect of interest in the population (effect size) necessary to calculate the sample sizes is still unknown. The power calculation is based on two assumptions; 1) the effect is indeed present in the data, and 2) the test statistic follows a normal or near normal (Student‘s distribution) α=0.05 and 1-β=0.8.The power calculations were done using Minitab V.18, State College PA.

**Supplementary Method S1**

Lipidomic analyses were performed at the West Coast Metabolomics Center (Davis, CA, USA), a National Institute of Health Regional resource core (http://metabolomics.ucdavis.edu/).

**Extraction method**

Extraction was carried out using a bi-phasic solvent system of cold methanol, methyl *tert*-butyl ether (MTBE), and water. In more detail, cold methanol (225 µL) containing a mixture of odd chain and deuterated lipid internal standards [lysoPE(17:1), lysoPC(17:0), PC(12:0/13:0), PE(17:0/17:0), PG(17:0/17:0), sphingosine (d17:1), d_7_-cholesterol, SM(17:0), C17 ceramide, d_3_-palmitic acid, MG(17:0/0:0/0:0), DG(18:1/2:0/0:0), DG(12:0/12:0/0:0), and d_5_-TG(17:0/17:1/17:0)] was added to a 20 µL plasma or 20 µL urine aliquot, which was placed into a 1.5 mL Eppendorf tuve. The tube was mixed for 10 s. Then, 750 µL of cold MTBE containing CE(22:1) (internal standard) were added, followed by vortexing for 10 s and shaking for 6 min at 4ºC. Phase separation was induced by adding 188 µL of mass spec-grade water. After vortexing for 20 s the sample was centrifuged at 14,000 rpm for 2 min. The upper organic phase was collected in two 300 µL aliquots. One was stored at -20ºC as a backup and the other was evaporated to dryness in a SpeedVac. Dried extracts were resuspended using a mixture of methanol/toluene (9:1, v/v; 60 µL) containing an internal standard [12-​[[(cyclohexylamino)carbonyl]amino]-​dodecanoic acid (CUDA)] used as a quality control.

**Chromatographic analysis**

The LC-MS analyses were performed using an Agilent 1290 Infinity LC system (G4220A binary pump, G4226A autosampler, and G1316C Column Thermostat) coupled to an Agilent 6530 mass spectrometer. Lipids were separated on an Acquity UPLC CSH C18 column (100 x 2.1 mm; 1.7 µm) maintained at 65°C at a flow-rate of 0.6 mL/min. Solvent pre-heating (Agilent G1316) was used. The mobile phases consist of 60:40 acetonitrile:water with 10 mM ammonium formate and 0.1% formic acid (A) and 90:10 propan-2-ol:acetonitrile with 10 mM ammonium formate and 0.1% formic acid. The gradient was as follows: 0 min 85% (A); 0–2 min 70% (A); 2–2.5 min 52% (A); 2.5–11 min 18% (A); 11–11.5 min 1% (A); 11.5–12 min 1% (A); 12–12.1 min 85% (A); 12.1–15 min 85% (A). Injection volumen was 1.7 µL and 5 µL for plasma and urine samples, respectively. Sample temperature was maintained at 4°C in the autosampler. The quadrupole/time-of-flight (QTOF) mass spectrometer was operated with electrospray ionization (ESI) performing full scan in the mass range *m*/*z* 65–1700 in positive (Agilent 6530, equipped with a JetStreamSource). Instrument paramaters were as follows: Gas Temp 325°C, Gas Flow 8 l/min, Nebulizer 35 psig, Sheath Gas 350°C, Sheath Gas Flow 11, Capillary Voltage 3500 V, Nozzle Voltage 1000V, Fragmentor 120V, Skimmer 65V. Data (both profile and centroid) were collected at a rate of 2 scans per second.
